# Successful treatment of a Caucasian case of multifocal Castleman’s disease with TAFRO syndrome with a pathophysiology targeted therapy - a case report

**DOI:** 10.1186/2162-3619-4-3

**Published:** 2015-01-14

**Authors:** Silvia Tedesco, Laura Postacchini, Lucia Manfredi, Gaia Goteri, Michele M Luchetti, Antonella Festa, Armando Gabrielli, Giovanni Pomponio

**Affiliations:** Clinica Medica - Dipartimento di Scienze Cliniche e Molecolari, Università Politecnica delle Marche, Via Conca 71, 60126 Ancona, Italy; Anatomia Patologica - Dipartimento di Scienze Cliniche e Molecolari, Università Politecnica delle Marche, Via Conca 71, 60126 Ancona, Italy; Clinica Medica – Ospedali Riuniti di Ancona, Via Conca 71, 60126 Ancona, Italy

**Keywords:** Castleman’s disease, Multicentric, TAFRO syndrome, Tocilizumab, Rituximab, Chemotherapy, PRES

## Abstract

**Background:**

Castleman-Kojima disease (TAFRO Syndrome) is characterized by Thrombocytopenia, Anasarca, myeloFibrosis, Renal dysfunction, Organomegaly, multiple lymphadenopathy and histopathology pattern of atypical Castleman’s disease (CD). Only few cases of this recently identified unique variant of Multicentric CD (MCD) are described in literature, all Japanese. It therefore poses serious diagnostic and therapeutic challenges.

**Case description:**

We describe a 21 year old woman with fever, asthenia, bilateral pleural effusion, ascites, hypoalbuminemia, severe thrombocytopenia, anemia, renal failure and proteinuria, whereas microbiological tests, immune serology (except ANA) and bone marrow biopsy were all negative. A CT-scan showed multiple lymphadenopathy and tissue samplings of mediastinal lymph nodes was compatible with a mixed-type CD. The diagnosis of MCD with TAFRO syndrome was made, but after an initial improvement with high dose corticosteroid therapy, clinical and laboratory features worsened. Based upon the high serum IL-6 levels and the high number of CD20-lymphocytes in lymph nodes tissue, we started tocilizumab (partial benefit), followed by rituximab combined with CVP (cyclophosphamide, vincristine and prednisone) chemotherapy, achieving a complete response. A total of six cycles of R-CVP were administered monthly, followed by maintenance with monthly rituximab. A complete remission persists at the 12th month of follow-up.

**Conclusions:**

In patients with massive immune system activation and lymphadenopathy it is mandatory to rule out Castleman-Kojima disease. In our patient a therapy aimed at the prominent pathophysiological abnormalities has been successful so far. However, since the rarity of TAFRO Syndrome, a multicenter registry is strongly desirable for a better understanding of the disease mechanisms, hopefully leading to evidence-based therapeutic choices.

## Background

Castleman-Kojima disease (TAFRO Syndrome) is a novel systemic inflammatory disorder characterized by a constellation of symptoms, namely, thrombocytopenia, anasarca, myelofibrosis, renal dysfunction and organomegaly, and multiple lymphadenopathy of mild degree with histopathology of mixed- or hyaline vascular-type Castleman’s disease (CD). This unique clinicopathologic variant of Multicentric CD (MCD) has been recently identified in Japan [1] and poses serious diagnostic and therapeutic challenges for pathologists and clinicians, including the differential diagnosis from autoimmune diseases.

Two are the main peculiarities of the case we herein describe: 1. This is the first report of a Caucasian patient; 2. The patient has been successfully treated with a combination therapy of immunosuppressive and cytotoxic drugs.

## Case presentation

### Case description

A 21 year old Caucasian woman with no relevant medical, family or psychosocial history was admitted to our department for pleural and pericardium effusion and ascites. The patient had been experiencing left subcostal pain, dyspnea and general malaise for four weeks before admission. She presented fever (38.5°C) with shiver, pharyngodynia, periorbital edema and asthenia (Table 1). One week before admission she visited a local hospital where she underwent a series of medical tests. Laboratory results revealed blood count abnormalities (thrombocytopenia and normocytic anemia), hypoalbuminemia, elevation of erythrocyte sedimentation rate (ESR), C-reactive protein (CRP), ferritin and γ-globulin (polyclonal) (Table 2). Abdominal ultrasonography showed hepato-splenomegaly with ascites, while a chest radiography and an echocardiography detected bilateral pleural and pericardial effusion. She was then referred to Ancona University Hospital for further diagnostic assessments.

**Table 1:** **Patient and disease characteristics at onset**

**Somatic features**	Caucasian female, 21 years old
**Genetic features and family history**	Negative
**Past medical history**	Isolated seizure in childhood (age 15)
	Car accident with right femur fracture (age 17)
**Signs and symptoms at onset**	Fever (38.5°C) with shiver
	Asthenia
	General malaise
	Pharyngodynia
	Left subcostal pain
	Dyspnea
	Periorbital edema
**Abnormal laboratory data at onset**	White blood cells 11.8 × 10^3^/μL
	Hemoglobin 11.5 g/dL
	Platelet counts 29 × 10^3^/μL
	AST 56 IU/L
	Albumin 1.86 g/dL
	Proteinuria 0.47 g/24 h
	CRP 22.2 mg/dL
	Ferritin 715 ng/mL

**Reference ranges and abbreviations.** White blood cells: 4–10 × 10^3^/μL; Hemoglobin: 11.5-16 g/dL; Platelet counts: 150-400 × 10^3^/μL; AST (aspartate aminotransferase): 0–40 IU/L; Albumin: 3.7-5.5 g/dL; Proteinuria: < 0.15 g/24 h; CRP (C reactive protein): 0–0.6 mg/dL; Ferritin 12–180 ng/mL.

**Table 2:** **Laboratory data of the patient**

	At the onset of disease	On admission	At 2 months after the onset (before TCZ)	At 3 months after the onset (after TCZ)	At 4 months after the onset (before R-CVP)	At 9 months after the onset (after sixth R-CVP)	At 12 months after the onset
**White blood cells (×10** ^**3**^ **/μL)**	11.8	15.9	26.6	12.9	20.9	7.5	8
**Hemoglobin (g/dL)**	11.5	8.9	6.3	13.2	8	14.7	13.9
**MCV (fL)**	90.7	85	86	102	92	97.5	97
**Platelet counts (×10** ^**3**^ **/μL)**	29	7	11	84	16	295	279
**AST (IU/L)**	56	104	16	25	19	17	12
**ALT (IU/L)**	-	67	18	34	71	10	9
**LDH (IU/L)**	340	637	552	630	824	-	-
**Albumin (g/dL)**	1.86	1.87	2.2	3	2.4	3.83	3.87
**Creatinine (mg/dL)**	0.8	1.6	1.3	0.6	0.9	0.6	0.6
**Proteinuria (g/24 h)**	0.47	1.96	4.52	0.9	1.21	0.1	0
**CRP (mg/dL)**	22.2	28.3	17.2	0.1	5.1	0.1	0.1
**Ferritin (ng/mL)**	715	1097	1126	-	1427	294	281
**IL6 (pg/mL)**	-	19.4	8.8	1.5	2.3	3	1

**Reference ranges and abbreviations.** White blood cells: 4–10 × 10^3^/μL; Hemoglobin: 11.5-16 g/dL; MCV (mean corpuscular volume): 80–98 fL; Platelet counts: 150-400 × 10^3^/μL; AST (aspartate aminotransferase): 0–40 IU/L; ALT (alanine aminotransferase): 0–40 IU/L; LDH (lactate dehydrogenase): 0–450 IU/L; Albumin: 3.7-5.5 g/dL; Creatinine: 0.6-1.4 mg/dL; Proteinuria: < 0.15 g/24 h; CRP (C reactive protein): 0–0.6 mg/dL; Ferritin 12–180 ng/mL; IL6 (interleukin 6): 0-5-2 pg/mL); TCZ: tocilizumab 8 mg/kg; R-CVP: rituximab 375 mg/m^3^, cyclophosphamide 750 mg/m^3^, vincristine 1.4 mg/m^3^ and prednisone 40 mg/m^3^.

**Figure 1:**
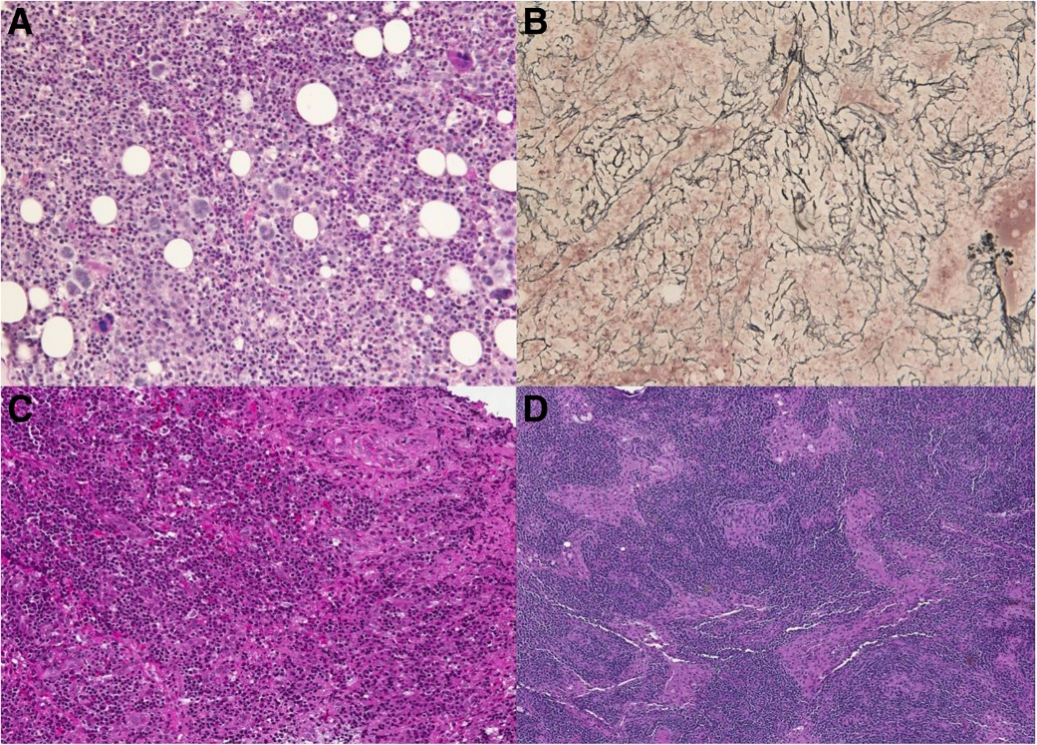
**Histopathological findings. (A)** The bone marrow appeared hypercellular with a marked expansion of granulopoiesis and a moderate increase of megakaryocytes (Periodic acid–Schiff (PAS), 20X). **(B)** On sections stained with the Gomori's silver impregnation technique, a diffuse and dense increase in reticulin with extensive intersections, occasionally with only focal bundles of collagen was evident. Fiber density was considered as grade 2 according to the European Consensus Criteria for grading myelofibrosis by Thiele et al. [2] (20X). **(C,D)** Multiple fragments of lymph node tissue were excised from the mediastinum of this patient. The architecture was preserved, showing CD20+ B-follicles and germinal centers with onion-skin appearance around prominent arterioles. The interfollicular areas showed abundant plasma cells with normal kappa:lambda ratio. Immunohistochemistry did not reveal aberrant B- or T-cell phenotype. Molecular testing was negative for B-cell or T-cell monoclonality. The search for HHV8 and EBV was also negative. The histology pattern was considered not diagnostic for malignant lymphoma and related to a lymphadenopathy with features resembling multicentric Castleman’s disease. The diagnosis was reviewed at the referral center of Bologna (Prof. Pileri) and confirmed.

**Figure 2:**
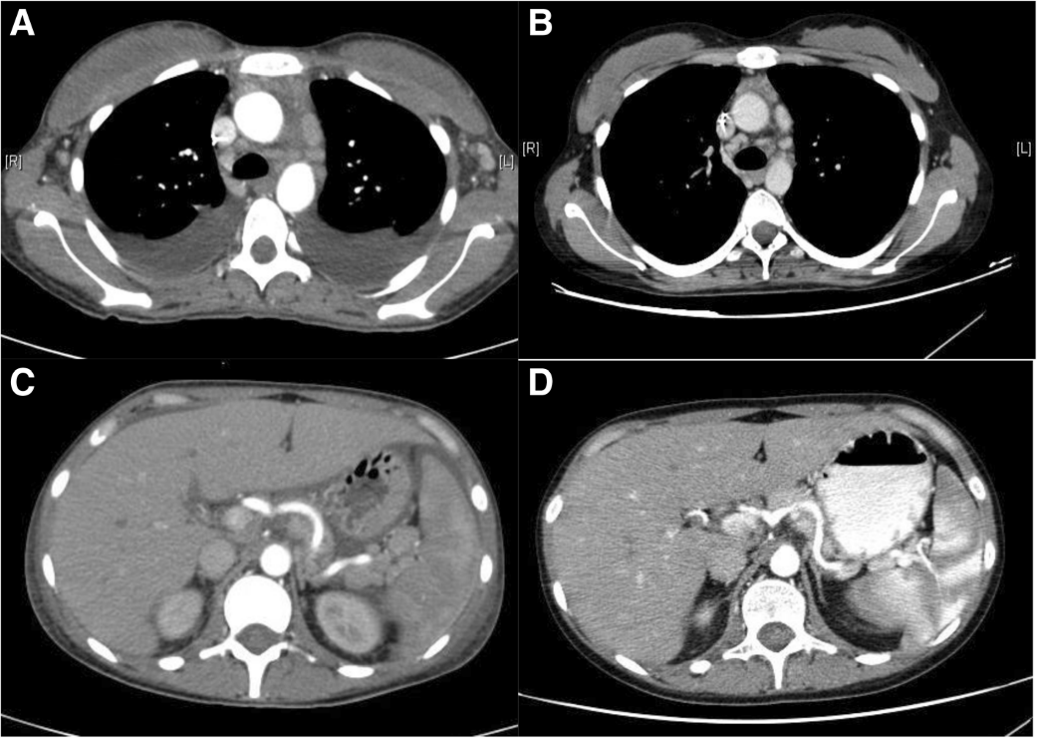
**CT images. (A, B)** Contrast enhanced thoracic computed tomography (CT) before **(A)** and after **(B)** six months of chemotherapy, showing resolution of pleural effusion and the shrinking of axillar and mediastinal lymphnodes. **(C, D)** Contrast enhanced abdominal CT before **(C)** and after **(D)** six months of chemotherapy, showing the reduction of hepato-splenomegaly and celiac and perisplenic lymphadenopathy.

On admission, she was dyspnoic and febrile (37.8°); a severe pitting edema was evident in both legs and the abdomen wall was markedly outstretched due to ascites. No skin lesions were visible. Her superficial lymph nodes were not palpable.Further laboratory tests revealed mild renal failure and significant proteinuria, whereas repeated blood, peritoneum liquid and urine coltural samples were sterile. Anti-Toxoplasma, anti-Bartonella, anti-Rickettsia antibodies and Widal-Wright test were all negative, as well as anti-HIV, anti-CMV, hepatitis C virus antibodies and hepatitis B surface antigen test. Moreover, PCR tests did not detect the presence of Epstein-Barr Virus DNA nor HHV-8 in the patient’s blood. Immune serology (anti-nDNA, anti-ENA, aCL, anti-β2GPI, ANCAs) were all negative. ANA were detected (1/640, granular pattern). The serum complement levels were normal. No monoclonal bands were observed on immunofixation tests. A bone marrow biopsy revealed a diffuse and dense increase in reticulin with reactive hyperplastic pattern (Figure 1A,B), and a mononuclear cells immunophenotyping was substantially normal. The serum level of IL-6 was significantly higher than normal: 19.4 pg/mL (reference range 0–5.2 pg/mL).After admission, a continuous fever ranging between 37.5° and 38°C persisted, and platelet, albumin and red blood cell transfusions were required. Additionally, the patient’s renal function rapidly deteriorated, with increased proteinuria. A whole body computed tomography (CT) showed bilateral axillar, mediastinal and abdominal para-aortic, celiac and perisplenic lymph nodes, hepatosplenomegaly, pleural and peritoneal effusion (Figure 2A,C).

The ascites and pleural fluids had biochemical exudative characteristics but were sterile, and no lymphoma or other malignant cells were detected in the samples.Tissue samplings of inguinal and mediastinal nodules (by bronchoscopy) were not diagnostic. After an initial improvement with high dose steroid therapy (methylprednisolone intravenously at 1 mg/kg for two weeks, then prednisone 50 mg/d orally, tapered to 37.5 mg/d), clinical and lab features worsened. A surgical mediastinal lymph node biopsy finally showed an histological picture suggestive for mixed-type (hyaline-vascular and plasma cell type) Castleman’s disease (Figure 1C,D).

The diagnosis of Multicentric Castleman’s Disease with TAFRO syndrome was then established.

Given the high plasmatic level of IL-6 and the clinical evidences available in literature, we added tocilizumab (8 mg/kg intravenously, every two weeks, three infusions) to the corticosteroid therapy (prednisone 50 mg/d orally); some of the patient’s features improved (Table 2), but after one month, there was no further clinic or laboratoristic benefit. Therefore, R-CVP (rituximab 375 mg/m^3^, cyclophosphamide 750 mg/m^3^, vincristine 1.4 mg/m^3^ and prednisone 40 mg/m^3^) chemotherapy was started (CVP monthly; rituximab weekly for the first month, then monthly). Two days after the first infusion a severe Posterior Reversible Encephalopathy Syndrome (PRES), clinically characterized by hypertension, visual disturbances, severe headache and convulsive crisis, appeared. A magnetic resonance imaging (MRI) of the brain showed enlargement of brain cerebrospinal fluid spaces and some small cortical-subcortical areas of altered signal. The syndrome resolved without any aftermath after three days. An anti-hypertensive and anti-comitial prophylaxis was added, the cyclophosphamide dose was reduced to 50% only in the second administration and the patient completed a total of 6 chemotherapy cycles without other adverse events.

**Figure 3:**
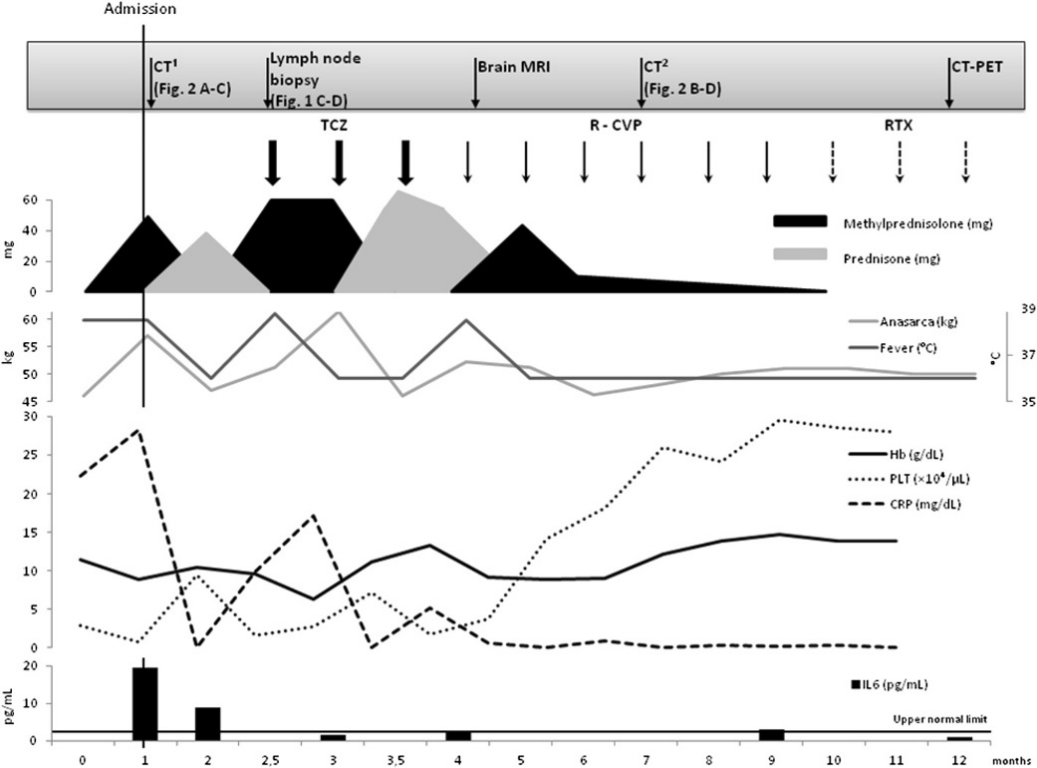
**Patient’s disease course (12 months-follow-up).** CT: computed tomography; MRI: magnetic resonance imaging; PET: positron emission tomography; TCZ: tocilizumab 8 mg/kg; R-CVP: rituximab 375 mg/m^3^, cyclophosphamide 750 mg/m^3^, vincristine 1.4 mg/m^3^ and prednisone 40 mg/m^3^; RTX: rituximab 375 mg/m^3^; Hb: hemoglobin; PLT: platelet counts; CRP: C reactive protein; IL6: interleukin 6.

Seven months after disease onset, a CT scan revealed no ascites or pleural effusion and reduction of lymphadenopathy (Figure 2B,D). After the sixth infusion (nine months from the disease onset), the patient’s symptoms had completely disappeared and blood tests, including the serum IL6 level, were within the normal range. Steroid therapy was carefully tapered during chemotherapy period and then discontinued. A single infusion of rituximab 375 mg/m^3^ is currently administered monthly as maintenance therapy.Twelve months after disease onset the patient continues to be totally asymptomatic and a 18-fluoro-deoxyglucose (FDG) positron emission tomography (PET) scan did not show any evidence of pathologic hyper-accumulation areas to be referred to disease activity. Clinical course and main diagnostic and therapeutic procedures are summarized in Figure 3.

## Discussion

**Figure 4:**
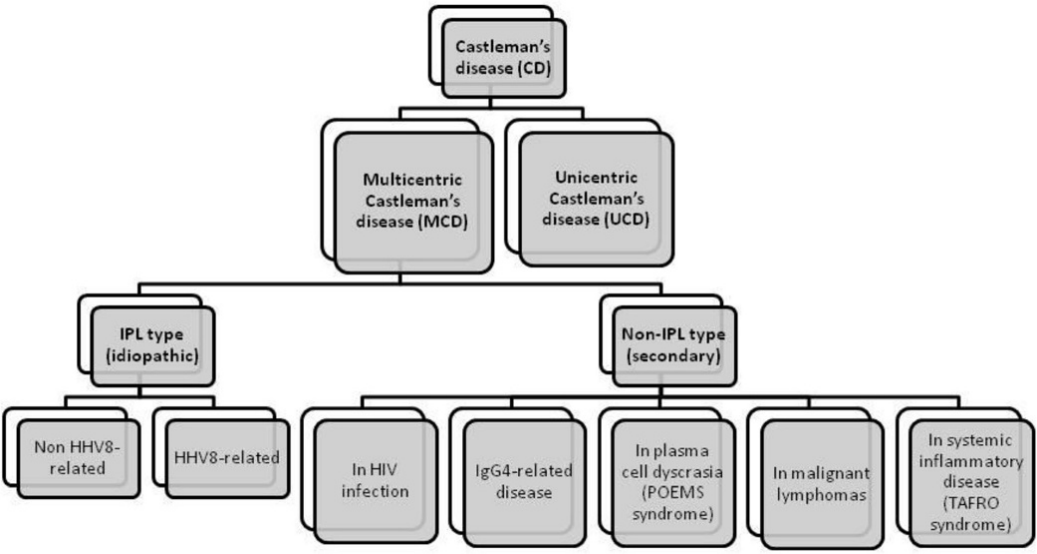
**New classification of MCD based on clinical and hystopatological features.** Modified from: Kawabata H, et al. Castleman-Kojima disease (TAFRO syndrome): a novel systemic inflammatory disease characterized by a constellation of symptoms, namely, thrombocytopenia, ascites (anasarca), microcytic anemia, myelofibrosis, renal dysfunction, and organomegaly : a status report and summary of Fukushima (6 June, 2012) and Nagoya meetings (22 September, 2012). J Clin Exp Hematop 2013, 53(1):57–61. IPL: Idiopathic Plasmacytic Lymphadenopathy; POEMS: Polyneuropathy, Organomegaly, Endocrinopathy/Edema, M-protein, Skin abnormalities; TAFRO: Thrombocytopenia, Anasarca, myeloFibrosis, Renal dysfunction and Organomegaly.

In a recent Japanese consensus conference [1], a new classification of MCD based on clinical and hystopatological features distinguished the Idiopathic Plasmacytic Lymphadenopathy (IPL)-type, either HHV8 positive or negative, from the non-IPL variants. TAFRO syndrome, POEMS syndrome, HIV-associated CD, malignant lymphoma-associated CD and IgG4-related diseases are the main entities of the latter (Figure 4).

The TAFRO syndrome, that in the past may have been occasionally described under a MCD label (even in Caucasian patients [3]), is characterized by a constellation of symptoms resembling the most severe autoimmune diseases (Systemic Lupus Erythematosus -SLE- or systemic vasculitis) and because of the nonspecific manifestations at onset, a careful and prolonged follow-up is often needed to reach a definitive diagnosis and to start the treatment.

A review of the literature with sensible strategy was performed. Both PubMed and Embase databases were searched, with “tafro [All Fields] AND (“syndrome” [MeSH Terms] OR “syndrome” [All Fields])” and “Multi-centric Castleman’s Disease [Supplementary Concept]” as strategy. Manual search was added and 30 pertinent articles were found (last update July 2014). The research showed, after the identification of this unique variant of MCD, only a handful of TAFRO cases, all in Japan, treated with tocilizumab and/or rituximab and cyclosporine A [4–9], with discordant results. A standard therapy is therefore far to be established and a therapeutic strategy borrowed from the MCD experience is suggested [10].

Since the disseminated lymphadenopathy rarely enable complete surgical debulking [11, 12], patients with MCD always require systemic therapy [13]. Steroids have been commonly used, and a response rate of 60% has been achieved, although responses are transient [8].

Due to the role of IL-6 in the pathogenesis of CD, antibodies against its receptor have been used [14]. In several reported cases [6, 15, 16] tocilizumab was very effective: patients achieved a complete remission and the treatment was discontinued often without disease recurrence [17].

Moreover, anti-CD20 monoclonal antibody (rituximab) has increasingly been used as a front-line treatment in most chronic B-cell lymphoproliferative disorders, in combination with standard chemotherapeutic regimens. Some reports of its efficacy in MCD have been published [18, 19], both in HHV-8 negative and HHV-8 positive patients, alone [9] or in association with combined chemotherapy [20].

Finally, in MCD patients treated with lymphoma-based chemotherapy, such as cyclophosphamide, vincristine, doxorubicin, and either prednisone (CHOP) or dexamethasone (CVAD), the overall response rate is around 90%, with 50% complete responses, but relapses are common and the median survival around 19 months. Durable responses occur approximately in 25% of cases, and rare remissions have been sustained in excess of 15 years [21].

However, when to start chemotherapy, how many cycles are required and the role of an eventual maintenance therapy need to be further investigated.

In the present case, a pathophysiology-targeted treatment was chosen. Based upon the high IL-6 levels in the serum, steroid therapy was initially associated to tocilizumab. Because of the high number of CD20-lymphocytes in lymph nodes tissue, rituximab combined with CVP chemotherapy followed, obtaining a complete clinical and biological response. Furthermore, a maintenance therapy with rituximab 375 mg/m^3^ monthly has appeared to be effective and safe after a six months follow-up (first description in literature).

Moreover this case points out another rare condition, that is Posterior Reversible Encephalopathy Syndrome (PRES) [22, 23], a poorly understood and described clinical-radiological syndrome whose pathogenesis has been ascribed to altered cerebral circulation and endothelial dysfunction. Many immunosuppressive drugs, such as intravenous immunoglobulin, ciclosporin A, tacrolimus, interferon α and, as recently reported, cyclophosphamide, may be responsible for this syndrome. The most novel finding in recent clinical series is the high prevalence of autoimmune disorders, especially SLE [24]. Although PRES is not considered an autoimmune condition per se, the association with immunological diseases suggests that endothelial dysfunction may lie at the core of its pathophysiology. Further research is of course needed to assess the merit of this hypothesis.

## Conclusions

In conclusion, this is the first reported Caucasian case of MCD with TAFRO syndrome. To achieve a more precise definition of this novel entity, to establish criteria for diagnosis and to define a therapeutic strategy, as well as to better investigate the etiology of MCD also in non-Japanese patients, multicenter surveys are desirable. In the meantime, it is crucial that in patients with massive immune system activation and lymphadenopathy, without any known autoimmune diseases or other well-defined lymphoproliferative disorders, Castleman-Kojima disease should be suspected [5], especially if anasarca and ascites are present. In the absence of solid clinical evidence about efficacy of a specific therapeutic intervention, a pathophysiology-based treatment could be reasonable and effective.

## Consent

Written informed consent was obtained from the patient for publication of this Case report and any accompanying images. A copy of the written consent is available for review by the Editor-in-Chief of this journal.

## Abbreviations

CD: Castleman’s disease
MCD: Multicentric CD
TAFRO: Thrombocytopenia, Anasarca, myeloFibrosis, Renal dysfunction and Organomegaly
ECR: Erythrocyte sedimentation rate
CRP: C-reactive protein
ANA: Anti nuclear antibodies
anti-nDNA: Anti native DNA antibodies
anti-ENA: Anti extractable nuclear antigens antibodies
aCL: Anticardiolipin antibodies
anti-β2GPI: Anti beta2 glycoprotein I antibodies
ANCAs: Anti neutrophil cytoplasmic antibodies
CT: Computed tomography
R-CVP: Rituximab, methylprednisolone, cyclophosphamide, vincristine and prednisone
CVP: Cyclophosphamide, vincristine and prednisone
PRES: Posterior Reversible Encephalopathy Syndrome
MRI: Magnetic resonance imaging
FDG: 18-fluoro-deoxyglucose
PET: Positron emission tomography
IPL: Idiopathic Plasmacytic Lymphadenopathy
POEMS: Polyneuropathy, Organomegaly, Endocrinopathy/Edema, M-protein, Skin abnormalities
SLE: Systemic Lupus Erythematosus.
